# An Automated and Precise Approach for the Determination of Azide Residue in Angiotensin II Receptor Blockers Using In Situ Matrix Elimination Ion Chromatography with Switching Strategy

**DOI:** 10.3390/ijms26104895

**Published:** 2025-05-20

**Authors:** Chaoyan Lou, Shaojie Pan, Xiaolin Yu, Kaidi Zhang, Kai Zhang, Yan Zhu

**Affiliations:** 1 College of Quality and Standardization, China Jiliang University, Hangzhou 310018, China; 18868730104@163.com (S.P.); unffgg@163.com (X.Y.); 15098639069@163.com (K.Z.); 2 Ningbo Key Laboratory of Agricultural Germplasm Resources Mining and Environmental Regulation, College of Science and Technology, Ningbo University, Ningbo 315300, China; zhangkai1@nbu.edu.cn; 3 Department of Chemistry, Zhejiang University, Hangzhou 310028, China

**Keywords:** angiotensin II receptor blockers (ARBs), azide, in situ matrix elimination, ion chromatography (IC), column-switching strategy

## Abstract

Angiotensin II receptor blockers (ARBs), a critical class of second-generation antihypertensive drugs, require azide intermediates for constructing their biphenyl tetrazole pharmacophore. This synthetic reaction introduces hypertoxicity risks, as residual azides can induce fatal damage even at trace concentrations. The pharmacopoeias of most countries have highlighted the urgency for improved detection paradigms of the control of azides in ARBs. Current ion chromatography (IC) methods face analytical challenges due to matrix interference from organic solvents and incompatibility with hydrophobic ARB ingredients. Herein, an in situ matrix elimination ion chromatography methodology was established for the sensitive detection of trace azides in angiotensin II receptor blocker pharmaceuticals. The switching strategy used in the proposed methodology eliminates organic interference and avoids the incompatibility issue with ARB ingredients. Under the optimal conditions, the proposed method exhibited satisfactory linearity in the range of 2.0–200.0 ng/mL, with a correlation coefficient of 0.9996. Validation studies demonstrated a detection limit (LOD, S/N = 3) of 0.57 ng/mL and a quantification limit (LOQ, S/N = 10) of 1.89 ng/mL, surpassing the sensitivity requirements in pharmacopeias. Method robustness was confirmed, with recovery rates from 92.8 to 108.7% using spiked ARBs real samples, and the intra-day and inter-day RSDs were less than 9.7%. The proposed approach establishes a reliable, precise, and sensitive alternative for monitoring azide impurities in ARBs, and such a framework can overcome limitations such as solubility issues, contributing to a universal applicability to diverse hydrophobic drugs.

## 1. Introduction

Cardiovascular diseases (CVDs) are regarded as the leading cause of mortality and disability worldwide, and have raised escalating public health concerns over recent decades [1,2]. According to the World Health Organization (WHO), CVDs account for over 18 million annual deaths globally, representing more than 30% of total noncommunicable disease mortality. Among all the cardiovascular diseases, hypertension is the most prevalent and risky [3]. Notably, hypertension underlies 62% of stroke cases and 49% of myocardial infarction incidents, surpassing the disease burden attributed to malignancies and other chronic disorders. In China alone, the hypertensive population has reached an estimated 330 million, with an annual growth rate of 3% [4]. Clinical evidence demonstrates that sustained reductions in systolic blood pressure by 2 mmHg are associated with a 10% decline in mortality, highlighting the urgent need for timely and effective hypertension management.

**Figure 1:**
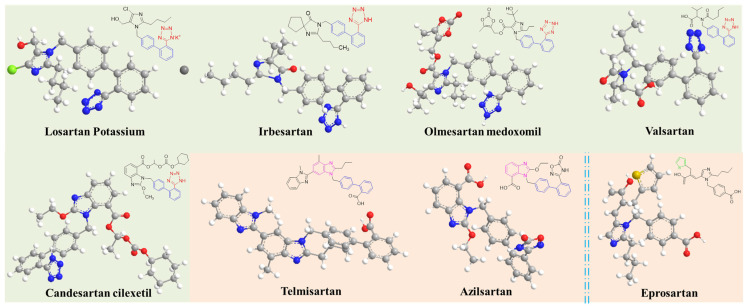
Chemical structures of various angiotensin II receptor blockers. ARBs with green background represent biphenyl tetrazole derivatives, and ARBs with red background represent non-biphenyl tetrazole derivatives. Specifically, telmisartan and azilsartan are typical benzimidazole sartan-class drugs, while eprosartan is a kind of heterocyclic sartan-class drug.

In antihypertensive therapy, angiotensin II receptor blockers (ARBs) have supplanted angiotensin-converting enzyme inhibitors (ACEIs) in many clinical scenarios due to their better tolerability [5]. Angiotensin II receptor blockers are second-generation antihypertensive therapeutics. Known as sartan-class drugs, ARBs exert their therapeutic effects by competitively binding to AT1 receptors on vascular smooth muscle cells, thereby antagonizing the vasoconstrictive effects of angiotensin II [6,7,8]. This mechanism reduces peripheral vascular resistance and achieves sustained blood pressure control [9]. These drugs demonstrate prolonged and stable antihypertensive efficacy, effectively reversing hypertension-induced target organ damage while reducing cardiovascular morbidity [10]. As a consequence, ARBs have been regarded as first-line therapeutics for long-term hypertension management due to their superior tolerability, minimal adverse reactions, and enhanced bioavailability. At present, ARB antihypertensives are primarily divided into several main categories due to their chemical structures (Figure 1): (a) biphenyl tetrazole derivatives (e.g., losartan potassium and irbesartan); (b) non-biphenyl tetrazole derivatives including benzimidazole sartan-class drugs (e.g., azilsartan and telmisartan), and other heterocyclic sartan-class drugs (e.g., eprosartan).

**Figure 2:**
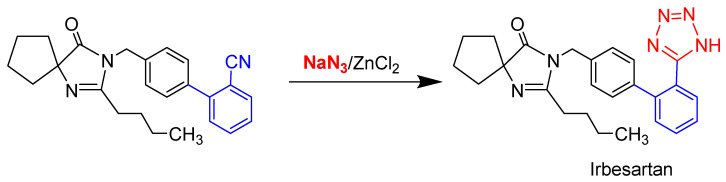
Synthesis of biphenyl tetrazole-based angiotensin II receptor blockers using azide reagents to generate the tetrazole pharmacophore in the final step.

It is of great importance to control the quality of angiotensin II receptor blockers because they are often used as long-term therapeutic antihypertensive drugs for patients. The synthesis of biphenyl tetrazole-based ARBs typically involves 5–10 reaction steps, with azide reagents (e.g., tributyltin azide and sodium azide) frequently employed in the final step to construct the tetrazole pharmacophore (Figure 2). However, azides, which are classified as highly toxic substances, pose severe health risks due to their rapid absorption via respiratory, digestive, or dermal routes, potentially causing fatal intoxication. Mechanistic studies indicate that azides inhibit cytochrome oxidase and other critical enzymes, disrupting oxyhemoglobin formation while exhibiting mucosal irritation, hypotensive effects, neurotoxicity, and potent mutagenicity [11]. Given these severe hazards, the strict regulation of azide residues in ARBs is imperative [12]. Major pharmacopoeias, including the Chinese Pharmacopoeia (Ch.P 2020), European Pharmacopoeia (EP 8.0), and United States Pharmacopeia (USP35-NF30), mandate an azide content limit of ≤10 mg/L [13]. This threshold aligns with the International Council for Harmonisation (ICH) classification of azides as DNA-reactive (mutagenic) impurities, which may induce carcinogenesis through direct DNA damage even at trace levels. Given the abovementioned hypertoxicity of azide compounds, the development of precise, sensitive, and robust analytical methods for monitoring azide residuals in ARB drugs is crucial for medication safety and quality control.

Current methodologies for azide analysis encompass UV spectrophotometry [14,15], gas chromatography (GC) [16,17,18,19,20], high-performance liquid chromatography (HPLC) [13,21,22,23], and ion chromatography (IC) [24,25]. Traditional methods like UV spectrophotometry or volumetric analysis suffer from low sensitivity and matrix interference, and conventional chromatographic techniques like GC and HPLC often require laborious derivatization or extensive sample pretreatment. In recent years, IC has emerged as the preferred approach due to its exceptional selectivity for ionic species, high detection sensitivity, and environmentally friendly operation, with minimal toxic solvent consumption. Despite these advantages of IC, the direct analysis of azides in ARBs remains impracticable due to the poor solubility of these sartan-class drugs in aqueous media and severe matrix interference from biphenyl tetrazole-based active pharmaceutical ingredients.

**Figure 3:**
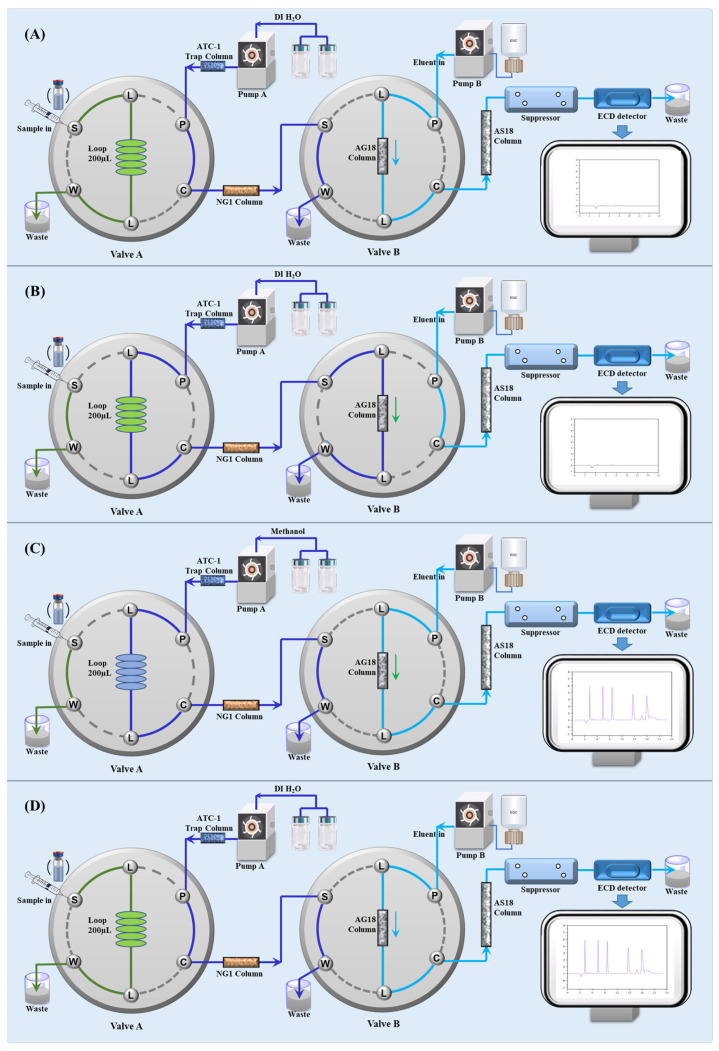
The operation scheme of in situ matrix elimination IC integration system. (**A**) Sample injection; (**B**) Matrix elimination and azide enrichment; (**C**) Analysis and detection; (**D**) System regeneration. To be specific, the green workflow represents the sample solution; the dark blue workflow represents eluent from Pump A; and the sapphire blue workflow represents mobile phase in IC system from Pump B.

To address the critical limitations of current analytical methodologies in analyzing hydrophobic ARB samples, this study established an integrated, in situ matrix elimination ion chromatography system for azide residue detection using a two-dimensional column-switching strategy. The automated flow path switching mechanism enables the direct analysis of hydrophobic ARB drugs (e.g., valsartan, candesartan cilexetil, and irbesartan) without further treatment, effectively overcoming the solubility constraints of existing ion chromatography. Leveraging the instrument’s inherent dual-column and dual-pump modules, this protocol avoids external switching interfaces and enables the full automation of sample cleanup without manual intervention (Figure 3A). This innovation significantly reduces artificial operation errors while enhancing throughput for batch testing in pharmaceutical quality control. This methodology establishes a feasible protocol for azide monitoring in hydrophobic ARBs, resolving the incompatibility of existing IC methods constrained by solubility issues. By aligning with pharmacopeial requirements, this validated approach turns out to be a robust, precise, and automatic framework for monitoring inorganic mutagenic impurities in ARB manufacturing.

## 2. Results and Discussion

### 2.1. Development of In Situ Matrix Elimination IC Integration System

#### 2.1.1. Operation Procedure

As described in Section 3.2, the ICS 5000 system is equipped with two valves and two pumps, which enables the direct integration of matrix elimination and analysis without external accessories or modules. As illustrated in Figure 3, the analytical protocol comprises four steps: (a) sample loading; (b) matrix trapping and azide enrichment; (c) separation and detection; and (d) regeneration.

Firstly, a 200 μL aliquot of the methanolic raw sample was injected into the loading loop in valve A (as shown in Figure 3A). Then, the sample was switched into the flow system by the pump (Pump A) connected with valve A. To avoid potential ionic contamination from the eluent, an ATC-1 trap column was occupied ahead of Pump A. In this step (Figure 3B), an eluent of deionized water was delivered by Pump A to flush out the methanolic solvent. The sample was sequentially directed through an NG1 column and an AG 18 column. The packing material of the NG1 column is a highly cross-linked macroporous copolymer with a very high hydrophobic surface area that exhibits poor retention of ionic compounds but has a strong retention of sartan-class molecules via reversed-phase interactions. Based on the mechanism, most of the organic matrix was trapped in the NG1 column, eliminating interference for the subsequent IC system. Azide ions were then flushed to the AG 18 anion-exchange concentrator column. The microspheres packed in the AG18 column area functionalized with quaternary ammonium groups; thus, there was a strong binding interaction between the azides and the microspheres. Simultaneously, Pump A flushed residual methanol to the waste container, ensuring compatibility with IC conditions. As depicted in Figure 3C, when Valve B switched to the injecting position, the ions were released into the ion chromatograph for further separation. In the meantime, the eluent of Pump A was changed to methanol to wash out the ARB active pharmaceutical ingredients retained in the NG1 column. Finally, the valves reverted to the initial condition to regenerate for next batch (Figure 3D).

#### 2.1.2. Optimization of Switching Time

The confirmation of the timing of valve-switching is essential for ensuring both analytical precision and operational efficiency in this automated system. As a key parameter, switching time not only determines the completeness of azide adsorption on the AG18 concentrator column but also impacts the total analysis time. To achieve a high recovery with minimal analysis time, the switching time was investigated from 0.0 to 3.0 min. The evaluation revealed a positive correlation between switching time and azide recovery (shown in Figure S1). The azide yield increased with time, and the maximum collection efficiency was achieved at 0.5 min. At the switching time of 0.5 min, the azide yield was sustained at a high level of 98%, and with a time extension, the azide yield was constant, indicating that the adsorption equilibrium was attained within 30 s. Consequentially, the switching time was set at exactly 0.5 min. Table 1 describes the specific operational instructions for the whole protocol.

#### 2.1.3. Optimization of Ion Chromatography Conditions

Given the anionic nature of azides (in the form of N_3_^−^), a hydroxide-selective anion-exchange system was adopted using an anion guard column (AG 18) and an anion analytical column (AS 18). These stationary phases were selected owing to their high selectivity so that azides could be separated from other common anions with similar retention behavior.

**Figure 4:**
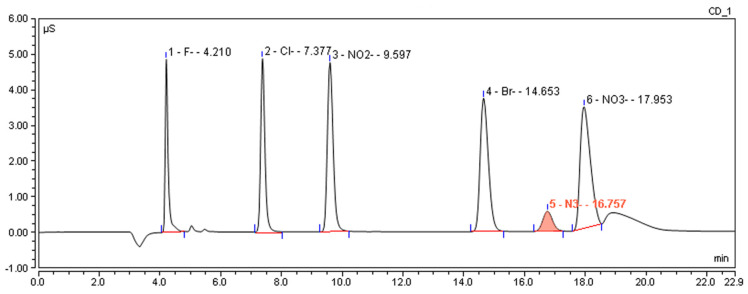
Chromatogram of azide and common anions under the optimal conditions.

Furthermore, a potassium hydroxide (KOH) gradient elution program was used as the mobile phase to achieve a better separation resolution as well as a shorter analysis time. An initial eluent concentration of 12 mM KOH (0.0–25.0 min) was used to effectively separate common inorganic anions from the azides. After that, the concentration of the eluent was increased to 30 mM (25.0–30.0 min) to flush out other potential strongly retentive ionic compounds. The columns were then regenerated with 12 mM KOH (30.0–35.0 min) to ensure system stability over consecutive injections. This gradient strategy achieved baseline separation of azides from other ionic compounds within 20 min and maintained symmetrical peak shapes across the linear range (Figure 4). Detailed experimental parameters as well as the operation procedure are depicted in Table 1.

### 2.2. Method Validation

**Figure 5:**
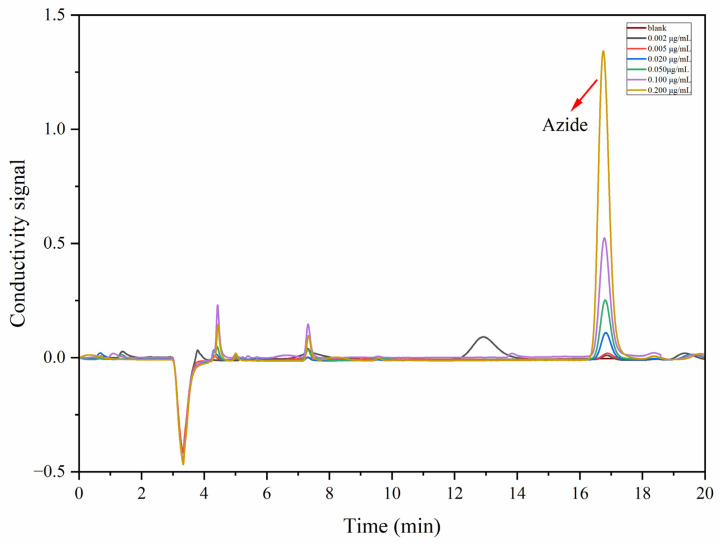
The representative chromatograms of standard samples at different levels.

The developed method was evaluated using validation tests, encompassing several analytical performance characteristics such as linearity, sensitivity, accuracy, and precision. In this research study, the azide content was quantified using an external standard method. The calibration curve was linear from 2.0 ng/mL to 200.0 ng/mL (six points), and their representative chromatograms are provided in Figure 5. The regression equation of the azides was calculated to be Y = 2.615X − 0.095, with a correlation coefficient of R^2^ = 0.9996. The sensitivity was investigated under the final conditions. The limit of detection (LOD), which was 0.57 ng/mL, and the limit of quantification (LOQ), which was 1.89 ng/mL, were calculated with signal-to-noise ratios of 3 and 10, respectively.

The precision and accuracy of the proposed method were validated via spiked recovery studies in sartan-class drug matrices. The spiked recovery of the method was examined at different spiked concentrations of 10 ng/mL and 100 ng/mL. The results demonstrated the recovery was more than 92.8% for all samples, with an inter-day RSD of less than 8% and an intra-day RSD of less than 10%. Comprehensive validation data are summarized in Table 2. The validation results confirmed the suitability of this methodology for routine azide monitoring in sartan manufacturing, which exhibited good sensitivity and robustness with high throughput.

Moreover, the proposed methodology is comprehensively compared with reported methods in the literature, demonstrating unique superiorities for azide identification in ARBs (shown in Table S1). By incorporating in situ matrix removal with ion chromatographic separation, this approach dispenses with the complex sample pretreatment steps required for conventional methods. The system achieves rapid analysis while maintaining high sensitivity, enabling the reliable detection of trace impurities even in strongly hydrophobic drug matrices. These combined advantages provide a practical and efficient solution for routine quality control in pharmaceutical manufacturing.

### 2.3. Analysis of Azide Residues in Sartan-Class Drug Products

**Figure 6:**
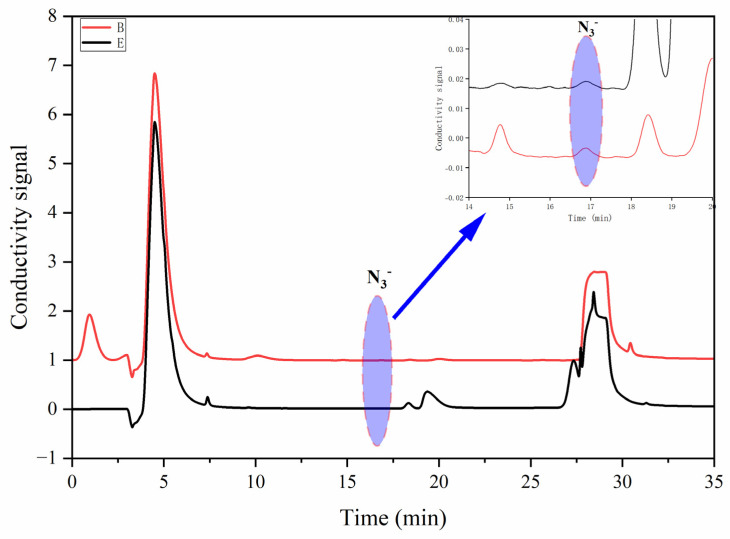
Representative chromatograms of two positive samples.

The proposed method was applied to the determination of azide residue in eight ARB samples (three candesartan cilexetil, two valsartan, two irbesartan, and one losartan potassium). Table 2 lists the results of azide contamination in eight real sartan samples, and Figure 6 describes some representative chromatograms of positive samples. In summary, while the levels of azide residue in the collected samples did not exceed pharmacopeial thresholds, the persistent detection of trace-level contamination underscores latent mutagenic risks associated with chronic exposure.

## 3. Materials and Methods

### 3.1. Chemicals and Materials

Due to the requirements for trace-level analysis, all the chemical reagents utilized in this work are chromatographic grade (≥99.9%, purity). The deionized water (resistivity of 18.2 MΩ·cm) was purified from a Milli-Q system (Millipore, Burlington, MA, USA). Sodium azide was purchased from Macklin Reagent Company (Shanghai, China) for preparation of azide stock solution and standard solutions. A five anion mixed standard in deionized water was purchased from Thermo Fisher Scientific (1000 mg/L). HPLC-grade methanol was purchased from Tedia Company (Johnstons, OH, USA). The 1000 mg/L azide stock solution was prepared by dissolving 0.0774 g of sodium azide in deionized water and diluting it to 50 mL in a volumetric flask, and the solution was stored in a laboratory refrigerator at 4 °C. A series of standard solutions used to create the calibration curve was prepared by diluting the stock solution. Membrane filters used for filtration were purchased from Thermo Fisher Scientific (Waltham, MA, USA).

### 3.2. Instrumentation and Equipment

The qualitative and quantitative analyses of azides were conducted in situ on a Thermo Fisher ICS 5000 system (Waltham, MA, USA), which was equipped with two quaternary pumps, an EG50 KOH eluent generator, an AERS500 anion suppressor (4 mm), a DS50 conductivity detector, and a Chromeleon Workstation. A Dionex IonPac AS18 column (250 mm × 4 mm) and a Dionex IonPac AG18 column (50 mm × 4 mm) produced by Thermo Fisher Scientific were adopted as the anion exchange separation column and the anion exchange guard column, respectively. An ATC-1 trap column (50 mm × 4 mm) and a NG1 pretreatment column (50 mm × 4 mm) were also configured in the system to eliminate the potential interference introduced by the eluent and the samples. A Milli-Q system (Millipore, Burlington, MA, USA) was used for purifying water. Also, an SBL-5DTS ultrasonic homogenizer (Ningbo Scientz Biotechnology Co., Ltd., Ningbo, China) was used for sample treatment.

### 3.3. Preparation of Standard Solutions

In this research, the content of azide residue in drugs was calculated using the external standard method. The standard calibration curve was established via serial dilutions of the stock solution across six concentration levels (2.0–200.0 ng/mL). In this research study, the simulated matrix solvent was prepared by dissolving 1.0 g of blank ARB powder in 50 mL of methanol and was used to create the abovementioned standard solutions at different concentration levels. Each concentration level was analyzed in triplicate under the same analytical conditions.

### 3.4. Pretreatment of Real Samples

Various ARBs in the form of a powder or a pill were obtained from pharmaceutical companies. Several different types, including losartan potassium, irbesartan, valsartan, and candesartan, were collected. In this work, the selection of ARBs was focused on biphenyl tetrazole derivatives, which involved an azide addition reaction. ARBs were subjected to grinding to achieve micronized powder for further preparation. Since ARB drugs exhibit insolubility in aqueous media but can dissolve freely in organic solvents (e.g., methanol), methanol was employed as the solvent of choice for the preliminary dissolution of ARB drugs. A 1.0 g portion of the raw sample was accurately weighed using an analytical balance and was quantitatively transferred to 50 mL volumetric flasks. During the procedure, the sample solution was sonicated in an ultrasonic homogenizer to ensure thorough dissolution. After that, the solutions were filtered through hydrophobic membrane filters (pore size 0.22 μm) prior to injection into the in situ matrix elimination IC integration system.

### 3.5. Incorporation of In Situ Matrix Elimination IC Integration System

In this work, the protocol of the automated matrix elimination ion chromatography integration system was fabricated in situ, without using any external modules or interfaces. Using the dual valves and dual pumps in ICS 5000, the whole system can be configured. As shown in Figure 3A, the whole system consisted of two valves, two pumps, an ATC-1 trap column, an NG1 column, and a routine ion chromatography system with suppressed conductivity detection.

### 3.6. Chromatographic Conditions

After optimization, the final ion chromatography conditions were as follows: The stationary phases were a Dionex IonPac AS18 column plus a Dionex IonPac AG18 column at a column temperature of 30 °C. The volume of injection was maintained at 200 μL. Suppressed conductivity detection was preferred in the analysis of trace azides due to its superior sensitivity for ions. As for the mobile phase, a gradient elution method was set up to ensure a thorough and resolved analysis. Specifically, during the first 25 min, 12 mmol/L of KOH eluted from the system. Then, 40 mmol/L KOH was used between 25 and 30 min. Subsequently, the concentration of KOH was adjusted back to 12 mmol/L for regeneration and for the next batch.

### 3.7. Validation Experiments

The developed methodology was rigorously assessed in accordance with regulations, including linearity, accuracy, robustness, and precision. A six-point azide calibration curve was generated across the range of 2.0–200.0 ng/mL, and each concentration level was tested in triplicate. The limit of detection (LOD) and the limit of quantification (LOQ) were calculated with signal-to-noise ratios of 3 and 10, respectively. The precision was evaluated using intra-day (n = 5) and inter-day (n = 3, over 72 h) relative standard deviations. Additionally, accuracy was confirmed via spiked recovery studies at concentrations of 10 and 100 ng/mL, respectively.

## 4. Conclusions

In this work, an in situ matrix elimination combined with ion chromatography was proposed and applied for the sensitive analysis of azide impurities in angiotensin II receptor blockers, especially biphenyl tetrazole derivatives such as valsartan and irbesartan. By designing a two-dimensional column-switching strategy, the proposed method successfully addressed the inherent incompatibility between conventional ion chromatography and the active, hydrophobic pharmaceutical ingredients of sartans. The system effectively eliminated organic matrix interference while selectively enriching trace azide ions in ARBs, thereby preventing column degradation and ensuring analytical reliability. Under the optimal conditions, this method demonstrated wide linearity, enhanced sensitivity, and distinguished accuracy. By aligning with pharmacopeial requirements, this approach established a robust framework for monitoring azide residue in ARB manufacturing. Moreover, this methodology exhibited broad and practical applicability across diverse drug formulations, overcoming the limitations of IC methods constrained by solubility issues.

## Figures and Tables

**Table 1:** Detailed procedure and operating parameters of the proposed method.

Steps	A	B	C	D
**Time/min**	/	0.0–0.5	0.5–30.0	30.0–35.0
**Valve A**	Load	Inject	Inject	Load
**Eluent A**	Deionized water	Deionized water	Methanol	Deionized water
**Valve B**	Inject	Load	Inject	Inject
**Eluent B**	12 mM KOH	12 mM KOH	12 mM KOH (0.5–25.0 min) 40 mM KOH (25.0–30.0 min)	12 mM KOH
**Statement**	Sample injection	Matrix elimination and azide enrichment	Analysis and detection	System regeneration

**Table 2:** Evaluation of accuracy and precision using spiked sartan drug samples.

Samples	ARBs	Added Levels (ng/mL)	Content of Azide Found ^a^ (ng/mL)	Recovery ^a^ (%)	Intra-Day RSD ^b^	Inter-Day RSD ^c^
A	Candesartan cilexetil	0.0	10.0 ± 2.1	/	4.3	7.1
		10.0	20.2 ± 3.0	101.8	3.5	8.3
		100.0	108.1 ± 4.9	98.1	4.9	6.0
B	Candesartan cilexetil	0.0	3.8 ± 1.9	/	5.5	7.5
		10.0	14.4 ± 2.5	105.5	6.4	7.2
		100.0	100.4 ± 3.4	96.6	6.7	6.8
C	Candesartan cilexetil	0.0	ND ^d^	/	/	/
		10.0	10.9 ± 2.7	108.7	5.3	9.7
		100.0	96.7 ± 5.3	96.7	6.7	7.6
D	Valsartan	0.0	7.3 ± 1.5	/	4.1	5.7
		10.0	17.8 ± 2.2	105.3	3.7	5.8
		100.0	105.8 ± 6.4	98.5	4.3	7.2
E	Valsartan	0.0	2.6 ± 1.6	/	5.5	7.1
		10.0	11.9 ± 1.8	92.8	4.1	8.6
		100.0	103.5 ± 5.2	100.9	5.9	7.9
F	Irbesartan	0.0	ND	/	/	/
		10.0	10.7 ± 1.4	107.2	4.4	8.1
		100.0	97.3 ± 3.5	97.3	5.8	8.3
G	Irbesartan	0.0	ND	/	/	/
		10.0	9.5 ± 1.7	95.4	4.7	7.7
		100.0	102.0 ± 4.2	102.3	7.2	8.6
H	Losartan Potassium	0.0	ND	/	/	/
		10.0	9.8 ± 2.4	97.8	5.3	8.2
		100.0	96.2 ± 4.5	96.2	6.7	7.9

^a^ The value of recovery represented average recovery. ^b^ The intra-day RSD was assessed using five consecutive injections within 24 h. ^c^ The inter-day RSD was assessed in triplicate across three independent days. ^d^ ND: not detected (<LOQ).

## Data Availability

Data is contained within the article and Supplementary Materials.

## Supplementary Materials

The following supporting information can be downloaded at https://www.mdpi.com/article/10.3390/ijms26104895/s1.

## References

[1] Munzel T., Hahad O., Sorensen M., Lelieveld J., Duerr G.D., Nieuwenhuijsen M., Daiber A. (2022). Environmental risk factors and cardiovascular diseases: A comprehensive expert review. Cardiovasc. Res..

[2] Shi Y., Zhang H., Huang S., Yin L., Wang F., Luo P., Huang H. (2022). Epigenetic regulation in cardiovascular disease: Mechanisms and advances in clinical trials. Signal Transduct. Target. Ther..

[3] Burnier M., Damianaki A. (2023). Hypertension as Cardiovascular Risk Factor in Chronic Kidney Disease. Circ. Res..

[4] Wang J.G., Zhang W., Li Y., Liu L. (2023). Hypertension in China: Epidemiology and treatment initiatives. Nat. Rev. Cardiol..

[5] Cutrell S., Alhomoud I.S., Mehta A., Talasaz A.H., Van Tassell B., Dixon D.L. (2023). ACE-Inhibitors in Hypertension: A Historical Perspective and Current Insights. Curr. Hypertens. Rep..

[6] Agostini L.D.C., Silva N.N.T., Belo V.A., Luizon M.R., Lima A.A., da Silva G.N. (2024). Pharmacogenetics of angiotensin-converting enzyme inhibitors (ACEI) and angiotensin II receptor blockers (ARB) in cardiovascular diseases. Eur. J. Pharmacol..

[7] D′Silva E., Meor Azlan N.F., Zhang J. (2022). Angiotensin II Receptor Blockers in the Management of Hypertension in Preventing Cognitive Impairment and Dementia—A Systematic Review. Pharmaceutics.

[8] Presta V., Figliuzzi I., Citoni B., Gallo G., Battistoni A., Tocci G., Volpe M. (2021). ARB-Based Combination Therapy for the Clinical Management of Hypertension and Hypertension-Related Comorbidities: A Spotlight on Their Use in COVID-19 Patients. High Blood Press. Cardiovasc. Prev. Off. J. Ital. Soc. Hypertens..

[9] Zhou Z., Orchard S.G., Nelson M.R., Fravel M.A., Ernst M.E. (2023). Angiotensin Receptor Blockers and Cognition: A Scoping Review. Curr. Hypertens. Rep..

[10] Seo S.M., Ihm S.H., Yi J.E., Jeong S.H., Kim B.S. (2022). Comparative efficacy and safety of fimasartan in patients with hypertension: A network meta-analysis of randomized controlled trials. J. Clin. Hypertens..

[11] Chourasiya S.S., Kathuria D., Kumar V., Ranbhan K.J. (2024). Mutagenic Azido Impurities in Drug Substances: A Perspective. Ther. Innov. Regul. Sci..

[12] Liu X., Hu T. (2021). Simultaneous Determination of Nitrite and Azide Ions in Valsartan. J. Chromatogr. Sci..

[13] Prihed H., Shifrovitch A., Shamai Yamin T., Madmon M., Belay C., Blanca M., Weissberg A. (2023). Rapid and simple identification of trace amounts of sodium azide in beverages and bodily fluids followed by derivatization and liquid chromatography-electrospray ionization tandem mass spectrometry. Rapid Commun. Mass Spectrom. RCM.

[14] Serratrice G., Béguin C., Chautemps P., Cogne C., Pierre J.-L. (2001). Biomimetic studies related to the azide-inhibited Cu,Zn superoxide dismutases. New J. Chem..

[15] Xue J., Luk H.L., Eswaran S.V., Hadad C.M., Platz M.S. (2012). Ultrafast infrared and UV-vis studies of the photochemistry of methoxycarbonylphenyl azides in solution. J. Phys. Chem. A.

[16] Bruin M.A.C., Dekker D., Venekamp N., Tibben M., Rosing H., de Lange D.W., Beijnen J.H., Huitema A.D.R. (2021). Toxicological analysis of azide and cyanide for azide intoxications using gas chromatography. Basic Clin. Pharmacol. Toxicol..

[17] Kudo K., Usumoto Y., Sameshima N., Okumura M., Tsuji A., Ikeda N. (2017). Reliable determination of cyanide, thiocyanate and azide in human whole blood by GC–MS, and its application in NAGINATA–GC–MS screening. Forensic Toxicol..

[18] Murakami T., Iwamuro Y., Sakamoto Y., Minami E., Ishimaru R., Tsuchihashi H., Chinaka S. (2023). Rapid Simultaneous Determination of Cyanide, Azide, and Ethanol in Whole Blood Using Headspace Gas Chromatography-Mass Spectrometry. Chromatographia.

[19] Pagliano E., Campanella B., D’Ulivo A., Mester Z. (2018). Derivatization chemistries for the determination of inorganic anions and structurally related compounds by gas chromatography—A review. Anal. Chim. Acta.

[20] Wachelko O., Szpot P., Zawadzki M. (2021). A novel simple and precise method for the determination of azide impurity in sartans using headspace gas chromatography with two dissimilar capillary columns and two flame ionization detectors (HS-GC-FID/FID). J. Pharm. Biomed. Anal..

[21] Gricar M., Andrensek S. (2016). Determination of azide impurity in sartans using reversed-phase HPLC with UV detection. J. Pharm. Biomed. Anal..

[22] Huang C.H., Tang M., Xu D., Shao B., Li P.L., Tang T.S., Qin L., Zhu B.Z. (2021). The critical role of unique azido-substituted chloro-O-semiquinone radical intermediates in the synergistic toxicity between sodium azide and chlorocatecholic carcinogens. Free Radic. Biol. Med..

[23] Jiang R., Xue X., Zhao F., Zhu W., Shang M., Su Y., Xu Y., Qian X. (2022). Process parameter and kinetic study for the azidation of a zidovudine intermediate with sodium azide in microreactors. Chem. Eng. J..

[24] Páll B., Gyenge Z., Kormány R., Horváth K. (2021). Determination of Genotoxic Azide Impurity in Cilostazol API by Ion Chromatography with Matrix Elimination. Separations.

[25] Zhang S., Han P., Xia Y. (2017). +Facile extraction of azide in sartan drugs using magnetized anion-exchange metal-organic frameworks prior to ion chromatography. J. Chromatogr. A.

