# Supplementary material for: An Automated and Precise Approach for the Determination of Azide Residue in Angiotensin II Receptor Blockers Using In Situ Matrix Elimination Ion Chromatography with Switching Strategy
Source: Int J Mol Sci. 2025 May 20;26(10):4895. doi: 10.3390/ijms26104895 (PMC12111811; doi:10.3390/ijms26104895)
Supplement: Supplementary file 1 [file ijms-26-04895-s001.zip › ijms-3661067-supplementary.pdf]

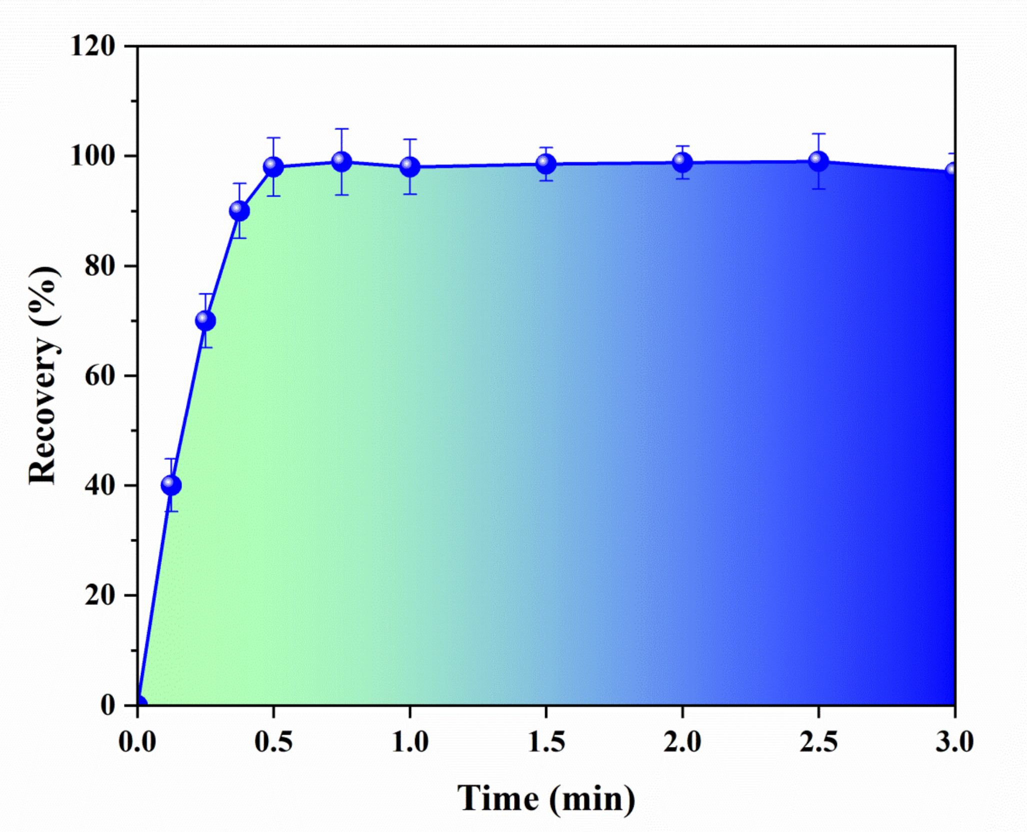

Figure S1. Effect of switching time on azide recovery.

Table S1. Comparison of the proposed approach with the major approaches reported in the literature.

Method	Target analyte	Matrix	Operation mode	Sensitivity (LOQ)	Recovery	Reference
**In-situ matrix elimination with IC**	**Azide**	**ARBs**	**Online**	**1.89 ng/mL**	**92.8%-108.7%**	**Proposed method**
GC-MS	Azide	Human whole blood	Offline	0.08 μg/mL	87%-96%	[17]
Headspace GC-MS	Azide	Whole blood	Offline	1.5 μg/mL	Not mentioned	[18]
Derivatization with LC/ESI-MS/MS	Azide	Beverages and bodily fluids	Offline	0.5 ng/mL	45%-57%	[13]
HPLC-UV	Azide	Sartan drugs	Offline	0.84 μg/g	94.0%-103.0%	[21]
LLE -IC	Azide	Cilostazol APIs	Offline	1.73 μg/mL	102.4%	[24]
MSPE-IC	Azide	Sartan drugs	Offline	0.79 ng/mL	96.5%-100.5%	[25]
